# Projecting the effects of climate change on *Calanus finmarchicus* distribution within the U.S. Northeast Continental Shelf

**DOI:** 10.1038/s41598-017-06524-1

**Published:** 2017-07-24

**Authors:** Brian D. Grieve, Jon A. Hare, Vincent S. Saba

**Affiliations:** 1 NOAA NMFS Northeast Fisheries Science Center, Narragansett, RI 02882 USA; 2 Integrated Statistics, Woods Hole, MA 02543 USA; 3 NOAA NMFS Northeast Fisheries Science Center, Woods Hole, MA 02543 USA; 4 NOAA NMFS Northeast Fisheries Science Center, Geophysical Fluid Dynamics Laboratory, Princeton University Forrestal Campus, Princeton, NJ 08540 USA

## Abstract

*Calanus finmarchicus* is vital to pelagic ecosystems in the North Atlantic Ocean. Previous studies suggest the species is vulnerable to the effects of global warming, particularly on the Northeast U.S. Shelf, which is in the southern portion of its range. In this study, we evaluate an ensemble of six different downscaled climate models and a high-resolution global climate model, and create a generalized additive model (GAM) to examine how future changes in temperature and salinity could affect the distribution and density of *C*. *finmarchicus*. By 2081–2100, we project average *C*. *finmarchicus* density will decrease by as much as 50% under a high greenhouse gas emissions scenario. These decreases are particularly pronounced in the spring and summer in the Gulf of Maine and Georges Bank. When compared to a high-resolution global climate model, the ensemble showed a more uniform change throughout the Northeast U.S. Shelf, while the high-resolution model showed larger decreases in the Northeast Channel, Shelf Break, and Central Gulf of Maine. *C*. *finmarchicus* is an important link between primary production and higher trophic levels, and the decrease projected here could be detrimental to the North Atlantic Right Whale and a host of important fishery species.

## Introduction

Generally speaking, zooplankton are ectothermic, short-lived, rarely exploited, and track environmental variation of water masses, making them good indicators of climate change^1^. Copepods are the most abundant zooplankton on earth, and one of the most abundant and widespread species on the Northeast U.S. Shelf is *Calanus finmarchicus* ^2^. *C*. *finmarchicus* ranges in small concentrations just north of Cape Hatteras, NC, USA up to larger concentrations in the Labrador Sea and the Northeast Atlantic^3^. In the Gulf of Maine, *C*. *finmarchicus* is a crucial primary consumer and a lipid-rich food source for a variety of important commercial fishery species, such as larval cod, haddock, and herring^4–6^. Concentrations of *C*. *finmarchicus* have also been linked to calving success of the critically endangered North Atlantic Right Whale^7–9^.

*Calanus finmarchicus* undergoes a complex life cycle consisting of six nauplii stages and five copepodite stages before reaching maturity^10^. During copepodite stage 5, *C*. *finmarchicus* in the Gulf of Maine enter diapause and overwinter at ~100–400 m, surviving on stored lipid reserves^11, 12^. In the spring, it comes out of diapause, moves towards the surface and reproduces, timing the end of diapause slightly before the onset of the spring phytoplankton bloom^13, 14^.

This reliance on multiple viable habitats potentially leaves *C*. *finmarchicus* particularly vulnerable to the effects of anthropogenic climate change^15^. In the Gulf of Maine, sea surface temperature has warmed three times faster than the global ocean average^16, 17^. This is especially concerning because *C*. *finmarchicus* on the Northeast U.S. Shelf is in the southern portion of its range, where environmental effects have a dominant role in shaping density and distribution^18, 19^. *C*. *finmarchicus* in the North Atlantic Ocean has already been observed shifting north at 8.1 km/year^20^. Climate change could also directly or indirectly affect egg production and hatching success of *C*. *finmarchicus* via temperature^21–23^ or acidification^24, 25^; however, these effects are still uncertain. The North Atlantic Oscillation also impacts population dynamics of *C*. *finmarchicus* ^26–28^, the intensity and frequency of which could be affected by a changing climate^29, 30^.

Due to the importance of *Calanus* species to their respective ecosystems, there have been multiple attempts to model their abundance. Some use detailed ecosystem processes to model present day populations^31–34^, while others use correlative and regression based statistical models^35–37^. Reygondeau and Beaugrand^38^ used an ecological niche model to project *C*. *finmarchicus* presence over the entire North Atlantic Ocean. They projected *C*. *finmarchicus* to be functionally extirpated (probability of occurrence <0.1) south of the Gulf of St. Lawrence by 2050–2059^38^. However, more knowledge on a finer geographic and seasonal scale is needed for the Northeast U.S. Shelf.

In order to examine how *C*. *finmarchicus* populations on the Northeast U.S. Shelf will be impacted in the future by climate change, we examined six climate models to determine how the fundamental niche of *C*. *finmarchicus* is expected to change with continued global warming. Using these projected temperatures and salinities, we created a generalized additive model (GAM)^39, 40^ that projects *C*. *finmarchicus* density over space and season through 2100. GAMs provide a flexible, nonparametric model that have frequently been used to project species density^41^, including *C*. *finmarchicus* ^20, 35^. Our approach can easily be applied to other marine species. Our projections are useful for fishery managers and conservationists as they consider the potential decline of a crucial primary consumer in their evaluations of predators’ present and future populations.

## Methods

To project future changes in *C*. *finmarchicus* density, we created a spatially and temporally resolved generalized additive model (GAM) following two different climate change scenarios. We used sampled density and environmental data, a species distribution model developed from those data, global climate model projections under two climate change scenarios, and a contemporary, regional climatology of temperature and salinity. We compared projections from coarse resolution climate models (~100-km ocean component) downscaled to a higher resolution climatology (~25-km ocean component) to projections made using a recently developed high-resolution global climate model (~10-km ocean component).

### *Calanus finmarchicus* and environmental data

**Figure 1:**
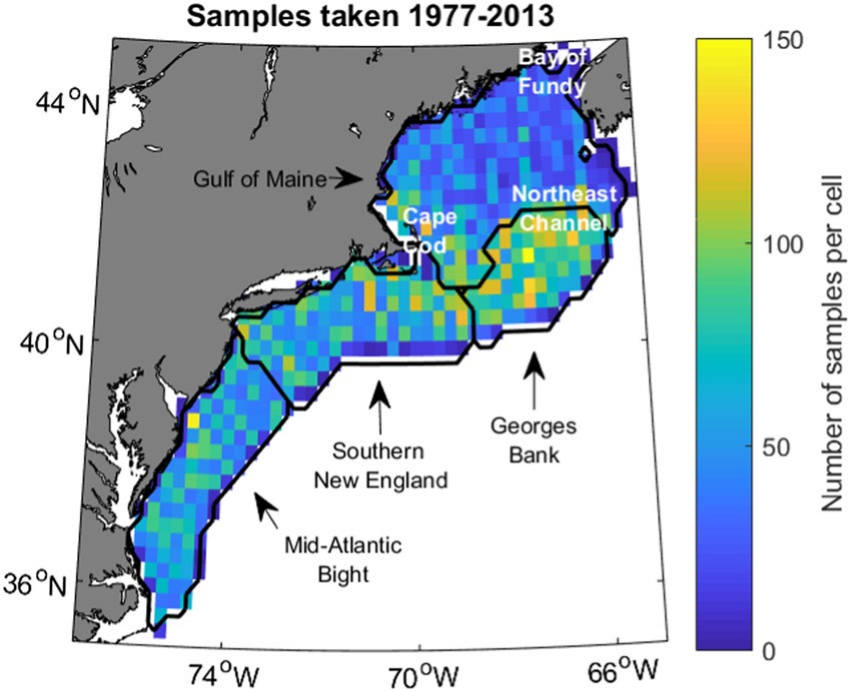
Number of samples taken in each 0.25° cell from 1977–2013. Black lines demarcate regions referred to in this paper, which are labeled by black arrowed text. White text marks locations of other features referenced. Figure was created by the first author in MATLAB v2015b (https://www.mathworks.com/products/matlab.html) using the package ‘M_Map’ v1.4 h (https://www.eoas.ubc.ca/~rich/map.html).

*Calanus finmarchicus* density data (n/100 m^3^) were obtained from NOAA NMFS surveys spanning 1977–2013. These surveys consisted of bongo tows (61-cm diameter) at depths of 200 m (or 5 m from the bottom) at stations across the Northeast U.S. Shelf^42, 43^. In the study region, only the central Gulf of Maine is deeper than 200 m. For each cruise, stations were fixed or randomly selected and spaced 8–35 km apart^43^. The mesh size (0.333 mm) reliably catches all *C*. *finmarchicus* in the life stages C3 and up, but not nauplii^44^. Tows were omitted from analysis if surface and bottom temperatures and salinities were not measured in conjunction with the tow. Based on these criteria, 17,159 samples were available for analysis from across the Northeast U.S. Shelf over 37 years. To better account for seasonal variability in *C*. *finmarchicus*, we categorized the data into six mean bimonthly “seasons” (January-February, March-April, etc.) and included season as a categorical variable in our model. The 17,159 samples and corresponding environmental variables were averaged into 0.25° bins for each season (N = 2654, 459–487 samples/season, median 6 samples/bin; Fig. 1). This ensured a balanced distribution of samples across the shelf, reducing spatial and temporal correlation of the model residuals. Geographic variability was analyzed by separating the Northwest Atlantic into four regions: the Gulf of Maine, Georges Bank, Southern New England, and the Mid-Atlantic Bight (Fig. 1).

### Species Distribution Model

To project future *C*. *finmarchicus* distributions and density, we used a generalized additive model (GAM)^40^ with a negative binomial distribution and a log-link function. GAMs model density using the cumulative smoothed response curves of each predictor variable to the response variable. GAMs are nonparametric, meaning that the relationship between the response and predictor variables is established by the data instead of by the modeler *a priori*. The binned data were not zero-inflated (~2%), however the large maximum values (4.5 × 10^6^ n/100 m^3^) caused a positive-skew and overdispersion (variance» mean). Both negative binomial distributions and the generalized modelling framework are adept at modelling the overdispersed data, so we chose not to log the response variable^45, 46^. The GAM was built in the R package ‘mgcv’^40, 47^.

Depth, sea surface temperature (SST), sea surface salinity (SS), bottom-water temperature (BT), bottom-water salinity (BS), and categorical bimonthly seasons were used as initial predictor variables for the model (Table 1). These variables were chosen because surface and bottom conditions could uniquely impact *C*. *finmarchicus* at different stages of its diapausal life cycle, are correlated with other potentially important variables such as nutrients and oxygen^48^, and are commonly modeled for multiple climate scenarios. Additionally, Albouy-Boyer *et al*.^36^ find that these variables, along with stratification and chlorophyll, were significant in modelling *C*. *finmarchicus* presence or abundance. We tested for multicollinearity using a Variance Inflation Factor (VIF) analysis for all variables^45^. Using 70% of the data, we compared all 63 possible combinations of the 6 variables in the GAM using AIC, deviance explained, and correct presence/absence classification percentage of the test data. This was repeated 100 times. Within the GAM, a Wald test determined whether the effect of a parameter is significantly different from zero. Although AIC was lowest using all variables, the effect of SST was weak (p = 0.053) and adjusted R^2^ was lower than with SST removed.

**Table 1:** Variables used to predict density in final generalized additive model.

Variable	Source	Average Spatial Resolution	p
Depth	ETOPO2	0.03° × 0.03°	≪0.01
SS	NOAA World Ocean Atlas	0.25° × 0.25°	≪0.01
BT	NOAA World Ocean Atlas	0.25° × 0.25°	≪0.01
BS	NOAA World Ocean Atlas	0.25° × 0.25°	≪0.01
Season	—	—	≪0.01
Climate Models	CCCma-CanESM2	0.90° × 1.40°	—
	CMCC-CMS	1.20° × 2.0°	—
	LASG-FGOALS-g2	0.90° × 1.0°	—
	JAMSTEC-MIROC5	0.80° × 1.0°	—
	MOHC-HadGEM2-CC	0.80° × 1.0°	—
	MPI-ESM-MR	0.50° × 0.50°	—

Model was trained using samples from NOAA surveys, and predictor variables used downscaled climate models. P-values are the result of a Wald test determining if smoothed model parameters are significantly different than 0.

The GAM was retrained with no subset. The final model took the form:

$$\begin{array}{c}{\rm{mean}}({\rm{density}}) \sim {\rm{s}}(\mathrm{ln}({\rm{depth}}))+{\rm{s}}({\rm{mean}}({\rm{SS}}))+{\rm{s}}({\rm{mean}}({\rm{BT}}))\\ \quad +\,{\rm{s}}({\rm{mean}}({\rm{BS}}))+{\rm{factor}}({\rm{season}})\end{array}$$

where s is a thin-plate regression spline. Maximum degrees of freedom for each smoother were set to 4 to avoid overly “wiggly” response curves that may not be ecologically meaningful. The penalty per degree of freedom in the smoothing parameter estimation function was multiplied by a constant 1.4, as suggested by Wood^40^ and Kim and Gu^49^, to avoid overfitting.

Density was determined by the GAM using predictor variables from each of the 6 climate models, utilizing both the historical and future RCP model scenarios. Historical projections were compared to observations to help evaluate the models.

### Climate Projections

We obtained ocean temperature and salinity projections from 6 general circulation models and earth system models used in the fifth Coupled Model Intercomparison Project (CMIP5) and the Intergovernmental Panel on Climate Change (IPCC) Fifth Assessment Report (AR5). These particular climate models (Table 1) were chosen because they are the only models currently available on the Earth System Grid Federation database that contain potential temperature and salinity over historical (1955–2005) and future (2006–2100) Representative Concentration Pathway (RCP) climate scenarios 8.5 and 4.5.

The RCP 8.5 is considered a “business as usual” scenario where greenhouse gas emissions, human population, and land use trends continue at roughly their current pace, beginning to level off by about 2100. RCP 4.5 is more “optimistic”, with greenhouse gas emissions tapering off by about 2050^50^. As of 2014, there has been little change in CO2 emissions from the RCP 8.5 path^51^.

Individual temperature and salinity climate projections were averaged into a single ensemble to remove internal variability and to recognize that ensemble averages are usually more accurate in recreating actual conditions than a single climate model by itself^52^. Depth was obtained from ETOPO2, a bathymetric representation of 2′ satellite altimetry (National Geophysical Data Center 2006). To match the resolution of other variables, the ETOPO2 bathymetry was spatially averaged into 0.25° bins. Depth was assumed to be consistent in all time periods and scenarios examined.

### Climatology and Delta Correction

Due to the relatively coarse resolution of the CMIP5 global climate models (~1° × 1°; Table 1), we downscaled the projections using the delta approach with a higher-resolution (~0.25° × 0.25° × 57 depth levels) climatology from the World Ocean Atlas (WOA). This climatology was constructed by the NOAA National Ocean Data Center from spatially interpolated observations, with the final product consisting of averaged ten year increments from 1955–2012^53–55^. The downscaling procedure takes the difference between the climate projections any given year and the initial climatology of that particular model, regrids it to the same resolution as the WOA climatology, and adds it to the WOA baseline. This technique preserves the resolved oceanographic features of the WOA dataset while still accounting for change indicated by the climate models^56^.

### GFDL CM2.6 Comparison

Downscaling using a high-resolution climatology is an improvement over the coarse model output, but still does not explicitly account for ocean dynamics at scales smaller than the coarse model resolution. The NOAA Geophysical Fluid Dynamics Laboratory (GFDL) has developed a high-resolution global climate model CM2.6^57, 58^, which resolves regional scale circulation in the Northeast U.S. that is not captured in coarse resolution models^17^. For example, CM2.6 (~10-km horizontal ocean resolution) projects a northwestern shift of the Gulf Stream and a poleward retreat of the Labrador Current, increasing the proportion of warmer and saltier Atlantic Temperate Slope Water entering the Gulf of Maine^17^. This model was only run using an 80-year simulation of the transient climate response (TCR), which projects a 1% per year increase in atmospheric CO_2_ such that CO_2_ doubles by year 70. This is not an IPCC RCP scenario, so it was not included in our ensemble projections of *C*. *finmarchicus*. However, it was still useful to compare the projections between the lower and higher resolution models. The CM2.6 TCR run can be equated to the IPCC’s RCP 8.5 scenario such that a doubling of CO_2_ (or a global surface warming of 2 °C relative to 1986–2005) is projected to occur in the 2060 s^59^. Therefore, the projections we show for CM2.6 can be roughly linked to the years 2050–2070 under the IPCC’s RCP 8.5 emissions scenario.

Using the same GAM trained with the samples, we projected *C*. *finmarchicus* density with variables from years 60–80 of GFDL CM2.6 and compared the projections to the ensemble projections at years 2050–2070. The downscaled climate ensemble was regridded to the same resolution as GFDL CM2.6 using nearest-neighbor interpolation to better compare the two resolutions and calculate a correlation coefficient between the two approaches. Because RCP 8.5 includes many other inputs and CO_2_ concentrations did not match up exactly with TCR, we did not necessarily expect *C*. *finmarchicus* projections to be the same. As such, we were primarily interested in changes in geographic patterns to determine where the lower resolution of the downscaled climate ensemble could have biased our results.

## Results

Sampled *C*. *finmarchicus* densities varied seasonally, annually, and spatially (Supplemental Figures S1 and S2). On average, *C*. *finmarchicus* was the most abundant during the spring and summer (March-August), with the peak density in May-June. The Gulf of Maine had the highest density, averaging 567,000 individuals (n) per 100 m^3^. *C*. *finmarchicus* was also common in Southern New England and Georges Bank during this period, averaging 210,000 n/100 m^3^. However there were very few *C*. *finmarchicus* in the Mid-Atlantic Bight (68,300 n/100 m^3^; Supplemental Figures S1 and S2). Sampled densities fell 57–78% during the fall and winter (September-February). Additionally, mean total Northeast U.S. Shelf *C*. *finmarchicus* density varied greatly from year to year, commonly halving or doubling from one year to the next (Supplemental Figure S1).

**Figure 2:**
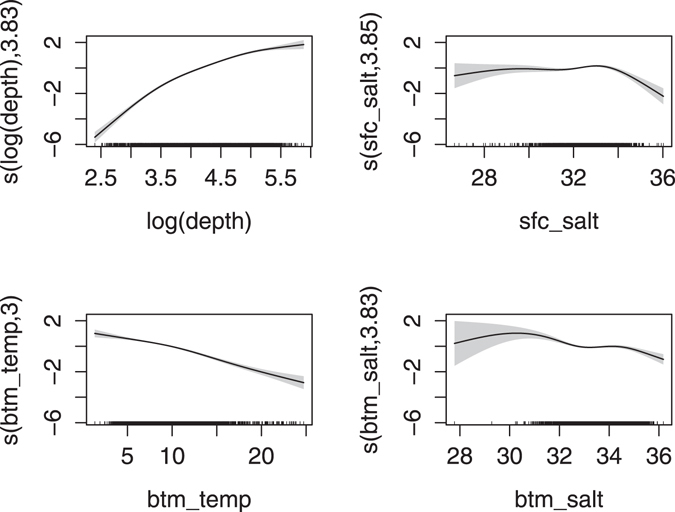
GAM response curves. Values indicate the relative impact of the variable on the projected population size, with shading indicating 2x standard error. Tick marks on the x-axis show individual observations. Numbers in the y-label indicate effective degrees of freedom for the variable.

The final GAM explained 64% of the deviance, had an adjusted R^2^ of 0.51 (N = 2654), and a correlation coefficient of 0.731 with the samples. This model represents the long-term average density of *C*. *finmarchicus* and does not attempt to capture the interannual variability. According to the Wald test, depth and bottom temperature were the most important predictive indicators. This is corroborated by the GAM response curves (Fig. 2). Depth is positively related to density up to 400 m, while BT is negatively related. Both bottom and surface salinity appear to have a negative influence when greater than ~34. Lower BS may be more beneficial. However despite minor multicollinearity (max VIF = 4.22) between these variables, all were still significant in the final GAM (p ≪ 0.01) after SST was removed. A variogram of the model pearson residuals showed very little spatial autocorrelation (Supplemental Figure S3).

The model was skillful in hindcasting the mean *C*. *finmarchicus* density during the sampling period, averaging 7% higher than the samples over all stations and seasons. The largest discrepancy between the observations and the model occurred in Southern New England, with the model averaging 31% lower than the observations (Supplementary Figure S4). Averaged over all seasons, Georges Bank was accurate (3% lower than observations), but is clearly overestimating September-December. Like the Mid-Atlantic Bight and Southern New England, the high percentages are caused by the very low sampled winter density. Averaged across all regions, the model was more accurate March-June (+8%), and was low in November-February (−20%). These seasonal averages are dominated by the Gulf of Maine, which is larger in area and initial density. The model overestimates (<10%) the central Gulf of Maine in March-April, and in the northeast in May-June (Supplementary Figure S4). All of the following results are in relation to the modeled present day density, and the observations are not considered further.

**Figure 3:**
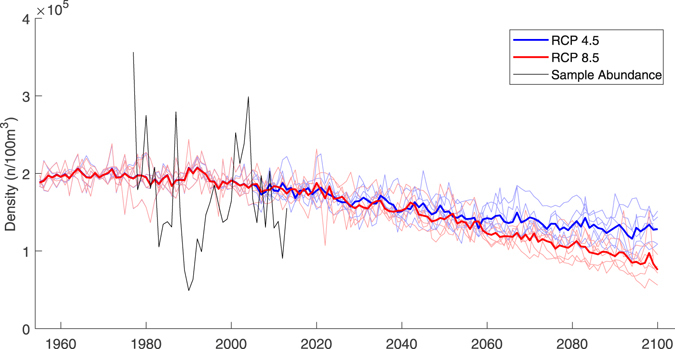
Projected density of *C*. *finmarchicus* on the Northeast U.S. Shelf. Individual climate model runs are indicated by thin colored lines while the ensemble average is bolded.

**Figure 4:**
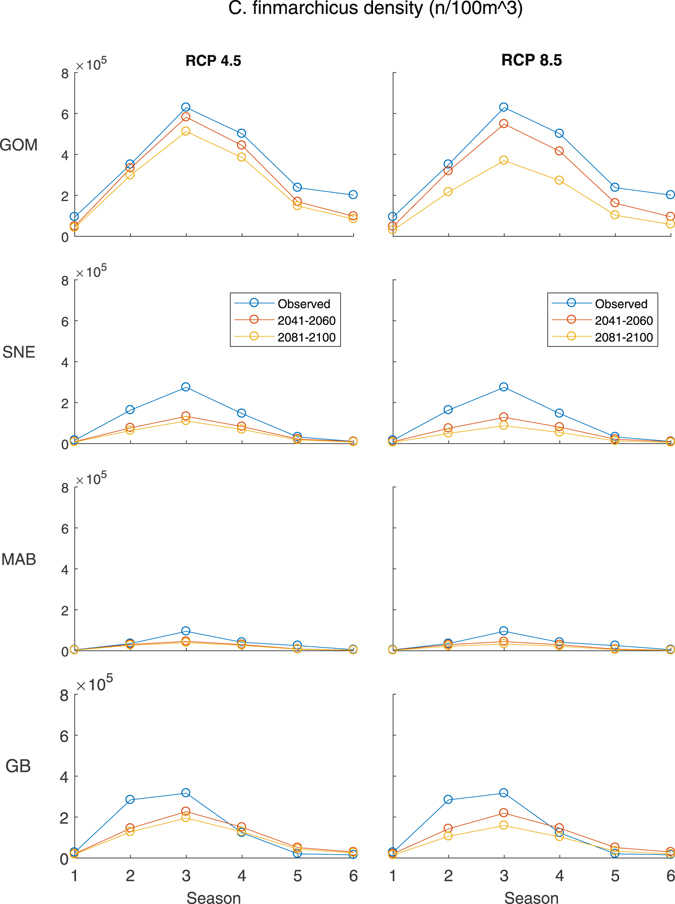
*C*. *finmarchicus* density (n/100 m^3^) in different regions, seasons, and RCP scenarios. The blue points are samples surveyed 1975–2013. Orange and yellow points are densities projected by the ensemble of climate models.

**Figure 5:**
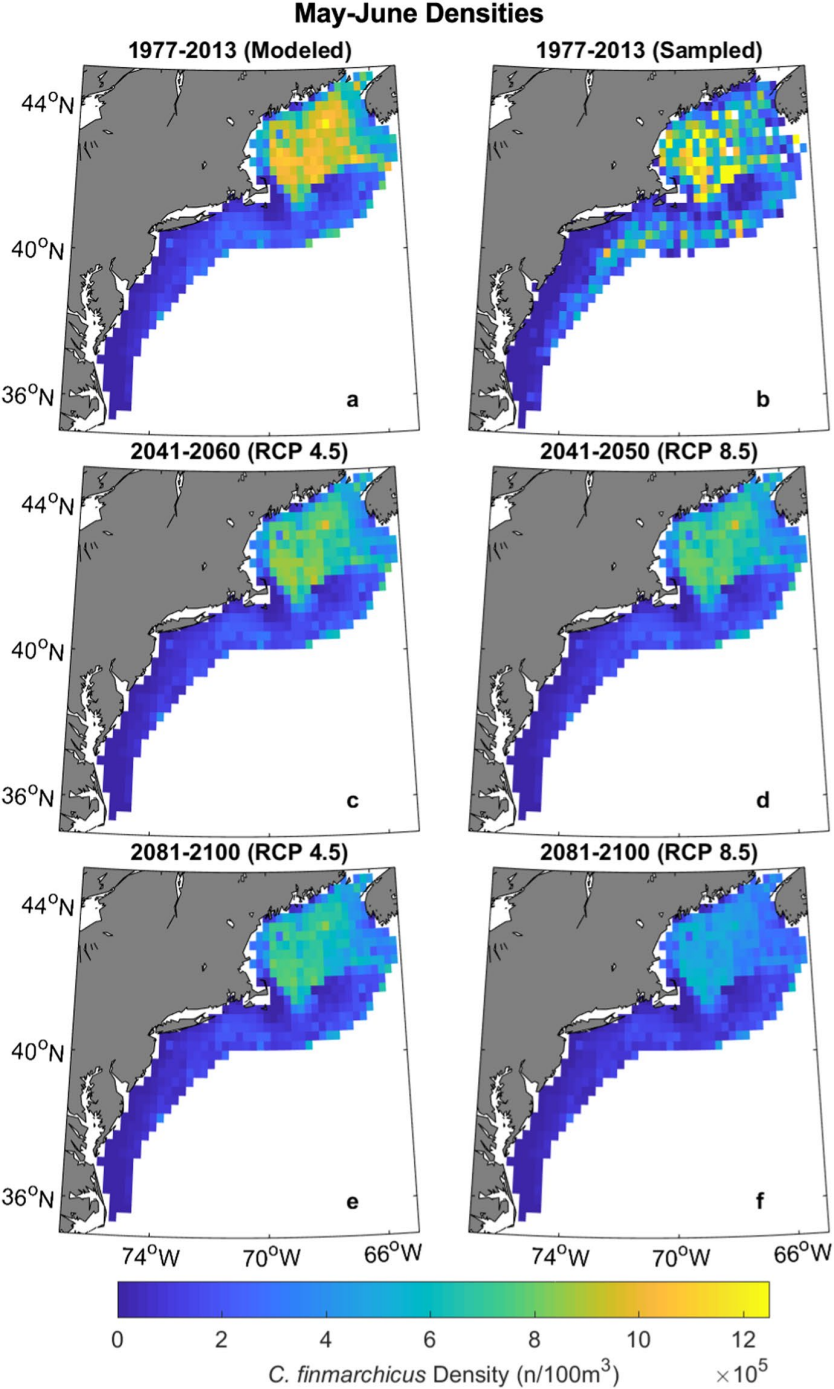
Projected May-June *C*. *finmarchicus* density over different time periods and climate scenarios. (**a**) Model hindcast under RCP 8.5. (**b**) Samples taken from 1977–2013. (**c**) Mean 2041–2060 density under RCP 4.5 and (**d**) RCP 8.5. (**e**) Mean 2081–2100 density under RCP 4.5 and (**f**) RCP 8.5. Figure was created by the first author in MATLAB v2015b (https://www.mathworks.com/products/matlab.html) using the package ‘M_Map’ v1.4 h (https://www.eoas.ubc.ca/~rich/map.html).

The trained GAM was used to project *C*. *finmarchicus* densities into the future under different climate scenarios. By the 2041–2060 period, there is expected to be similar decreases in *C*. *finmarchicus* density under the RCP 4.5 and RCP 8.5 scenarios, down 22% and 25% of present day density over all regions and seasons, respectively (Fig. 3, Supplementary Figures S5 and S6). After the 2041–2060 period, the two scenarios begin to diverge more quickly. By the 2081–2100 time period, the Northeast U.S. Shelf is projected to have 32% fewer *C*. *finmarchicus* under RCP 4.5 than present and 50% fewer under RCP 8.5 (Fig. 4, Supplementary Figures S7–S8). These declines were greatest during the peak density (May-June, Fig. 5), followed by the surrounding bimonthly seasons (Fig. 4). The peak density is projected to be 21% and 31% smaller under RCP 4.5 and RCP 8.5, respectively.

**Figure 6:**
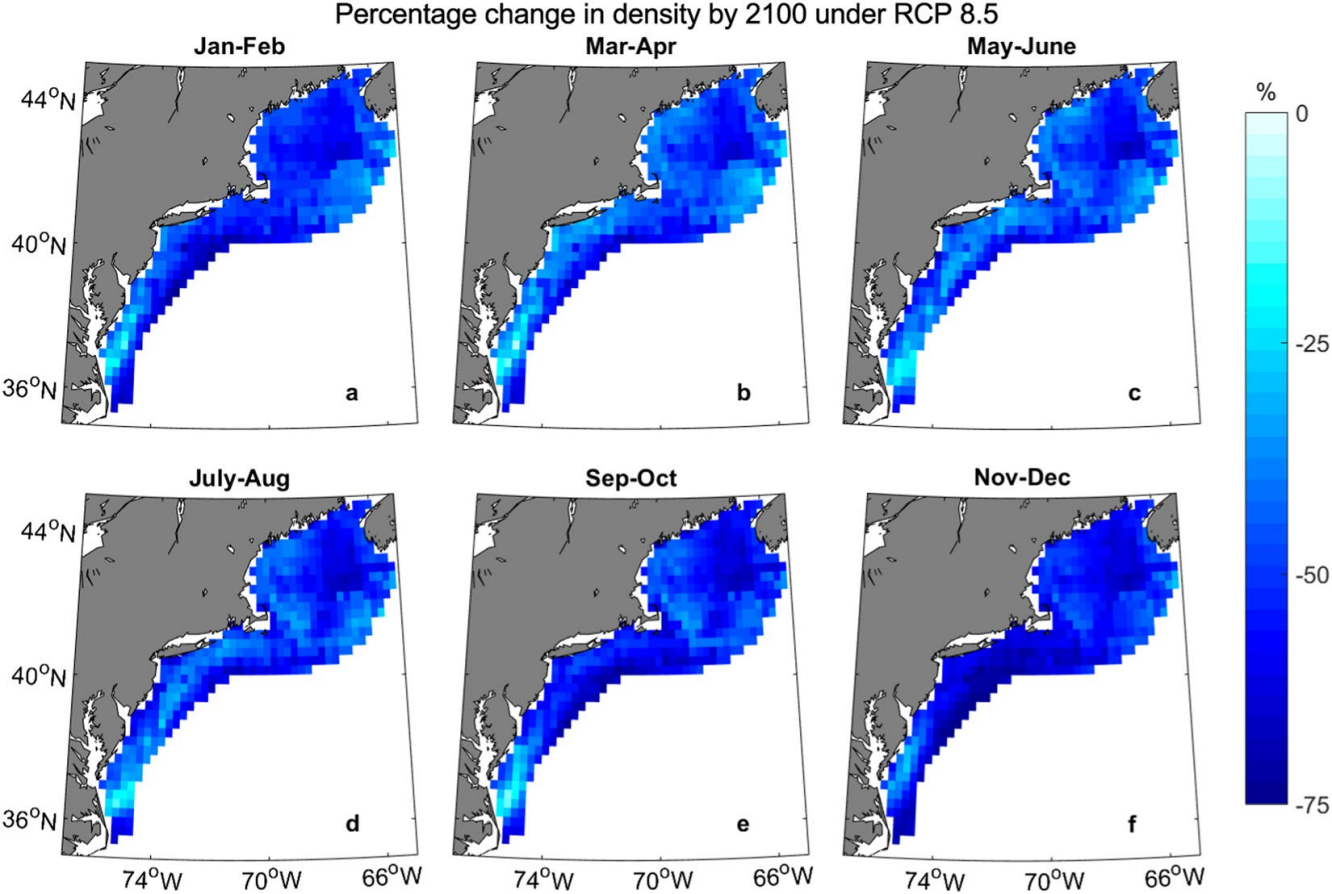
Percentage change in *C*. *finmarchicus* density between 2081–2100 and present period under RCP 8.5. Negative values indicate a decrease in density. White areas off the shelf were not included in this study. Figure was created by the first author in MATLAB v2015b (https://www.mathworks.com/products/matlab.html) using the package ‘M_Map’ v1.4 h (https://www.eoas.ubc.ca/~rich/map.html).

Total *C*. *finmarchicus* densities are expected to be most affected in the Gulf of Maine, particularly in the central Gulf and northeast (Figs 4–6, Supplementary Figures S5–S8). The shallower areas in the western Gulf may be less impacted, especially in the non-winter months; however losses are still expected for all scenarios, seasons, and time periods (Fig. 6). Conversely, eastern Georges Bank is not expected to lose a large amount of *C*. *finmarchicus*, while the central and western areas are more heavily impacted. *C*. *finmarchicus* decline in the Mid-Atlantic Bight and Southern New England is small compared to other regions, but proportionally greater due to the much smaller present-day populations, especially in the deeper areas (Fig. 6). These regions are impacted the most in the winter months and along the shelf edge. Despite being located at the trailing edge of the range, the southern Mid-Atlantic Bight has the smallest percentage decreases in the entire study area, losing ~20%. This may be misleading, as there is so few *C*. *finmarchicus* currently in the southern MAB, and that is one area the GAM hindcast was biased high (Supplemental Figure S4).

Six climate models were used for this analysis, each projecting historical, RCP 8.5, and RCP 4.5 conditions (Table 1). All of the climate models projected similar trends in density and none were obvious outliers (Fig. 3). The hindcasts from the individual models were similar, with a coefficient of variation (CV; standard deviation/mean) of 0.14. This is substantially smaller than the CV between individual years of the sampled time series (CV = 1.40). As expected, the models agreed less by 2081–2100 (Fig. 3). By 2081–2100, the RCP 8.5 models had a CV of 0.30 and the RCP 4.5 models had a CV of 0.25. For both scenarios, the standard deviation among the six models was highest in the central and western Gulf of Maine in May-August.

**Figure 7:**
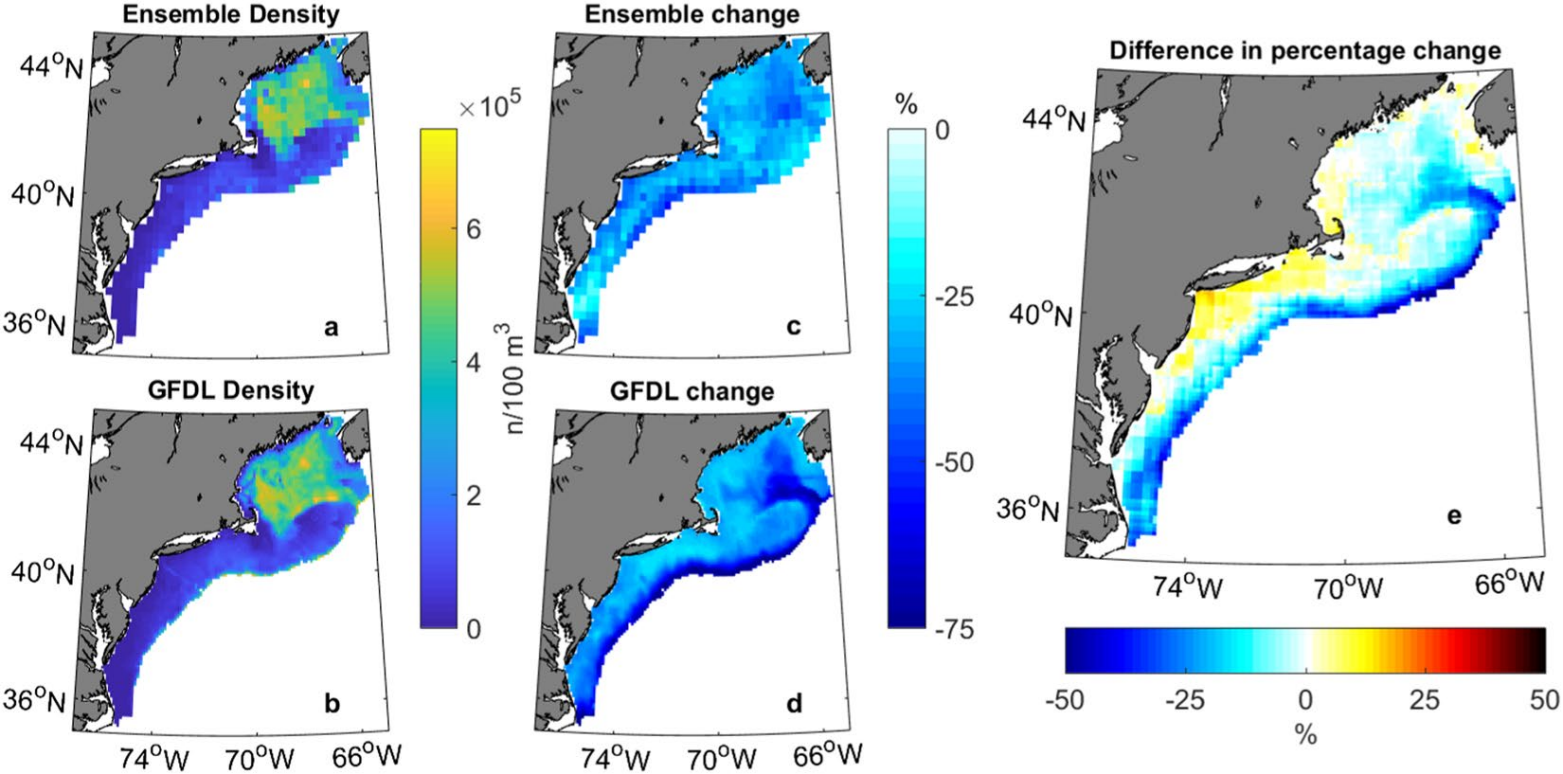
Comparison of the ensemble (0.25° RCP 8.5) and GFDL (.1° TCR). Time periods are twenty year annual averages for 1986–2005 of the historical ensemble scenario and 2041–2060 of RCP 8.5, compared to the same years 1–20 and 61–80 of GFDL CM 2.6 TCR scenario. (**a**) Early density of ensemble RCP 8.5 and (**b**) GFDL CM 2.6 TCR. (**c**) The percentage change between the late and early time periods of ensemble RCP 8.5 and (**d**) GFDL CM2.6 TCR. (**e**) The difference in the percentage change in density projected by each model (7d-7c). Warm colors indicate that the ensemble projects greater losses than GFDL in those regions. Correlation between the differences projected by the ensemble and GFDL CM 2.6 is 0.51. Figure was created by the first author in MATLAB v2015b (https://www.mathworks.com/products/matlab.html) using the package ‘M_Map’ v1.4 h (https://www.eoas.ubc.ca/~rich/map.html).

Despite the difference in forcings between RCP 8.5 and TCR, the two scenarios showed similar changes in space and density over all seasons. However, GFDL CM2.6 projected a less uniform change in density (Fig. 7). The largest discrepancy was on the shelf break and the Northeast Channel, which are both poorly resolved in the ensemble of coarse models, with GFDL CM2.6 projecting ~50% mean decrease in *C*. *finmarchicus* density and the ensemble projecting changes ~30% (Fig. 7). Based on this, it is also possible that the ensemble underestimates losses in the Central Gulf of Maine and overestimates losses in Southern New England, yet the projections from each method are still within 12% of each other. Overall, the total change in density between the two time periods in the ensemble and GFDL CM2.6 had a correlation coefficient of 0.51.

## Discussion

In this study, we modeled the density of the copepod *Calanus finmarchicus* with expected changes in water temperature and salinity with future climate change. We found that *C*. *finmarchicus* concentrations are projected to decrease across the Northeast U.S. Shelf, especially in the spring and summer months in the Gulf of Maine.

Overall, the species distribution model well represented the average seasonal and geographic patterns of *C*. *finmarchicus* density over the 37 year timeseries. The model fit (R^2^ = 0.51, 64% deviance explained), is good for this type of modeling, and is in the same range as similar models^35, 36, 38^. Model biases in the hindcast of mean *C*. *finmarchicus* density, such as overestimation in Georges Bank in winter or the Gulf of Maine in spring, may indicate that our projections are off by up to 10%. However, precise estimates (Supplementary Figures S5–S8) are not as important as the projected trend. If the model is consistent over time, the bias will cancel out of that trend.

The model did well at capturing long term averages of *C*. *finmarchicus* over space and season, but as expected, does not fit the substantial year-to-year variation of the observations. Neither the WOA climatology nor the climate models are designed to capture internal variability, and we spatially averaged all observations in the sampled timeseries into 0.25° bins before training the final model. Internal variability is projected in each climate model, however, it is not synchronized with present day conditions and the multi-model mean averages across the interannual variability. It is possible that indices of internal variability, such as the North Atlantic Oscillation, could have improved model fit to the individual sample years^27, 28^, but these indices would not be linked to modelled hydrography and are too difficult to reliably forecast in climate models to be useful future predictors. Advances in computational resources along with improved predictions of internal variability will greatly aid climate model projections of living marine resources, particularly for near-future management decisions^60^.

There are certain caveats and assumptions in this model. First, like most niche models, it does not include evolutionary adaption or localized phenotypic plasticity, and the response of the population to abiotic predictors is assumed to be constant. The impact of evolution on population size is impossible to predict, and *C*. *finmarchicus* connectivity across the North Atlantic is high, reducing genetically distinct local populations^61, 62^. Additionally, *C*. *finmarchicus* has thus far shown poor adaptation to increased temperatures in the Northeast Atlantic^63^. The model does not include species interactions. Species interactions may be more important than physical changes regarding distributions and climate change, however this effect is lessened for primary consumers and marine organisms^64^. Chlorophyll may be useful as a predictive measure in *C*. *finmarchicus* density and distribution models, but results are conflicting^20, 33, 38, 65^. Chlorophyll was not used in this study due to the limited number of presently-available earth system models that project potential temperature, potential salinity, and chlorophyll under multiple RCP scenarios. Ocean acidification may affect copepods at different stages of their life cycle, so pH could be considered as well^66, 67^.

Next, we do not explicitly include diapause and the seasonal energetics that contribute to *C*. *finmarchicus* role as a prey source^68, 69^. A more process-based species distribution model would be required to better capture the dynamics of diapause^61, 70^. Other abiotic metrics that have been used to project *Calanus* species density and distributions include surface stratification^36^ and currents and dispersal^31, 65, 71, 72^. However, stratification is a product of salinity and temperature used here, and IPCC projections of currents are unavailable at the resolution required to conduct a Lagrangian transport study.

Despite shortcomings of coarse climate models and simple delta downscaling, the comparison with high-resolution GFDL CM2.6 supports the accuracy of our methods here. The two models were within 12% of one another throughout the shelf; however, GFDL CM2.6 showed a more nuanced projection (Fig. 7). GFDL CM2.6 showed a larger decrease on the Shelf Break and the Northeast Channel where a northern shift in the Gulf Stream is unaccounted for in the ensemble of coarse climate models^17^. The ensemble projected greater losses in the Bay of Fundy, Southern New England, and around Cape Cod, likely due to the shallow bathymetry in these regions that were still not resolved in the downscaling.

Our results indicate climate change will cause large declines in *C*. *finmarchicus*, especially in the Gulf of Maine and Georges Bank. This decline is projected for both RCP scenarios at a similar rate through 2041–2060. After that, densities in the RCP 4.5 scenario decrease at a much slower rate, while populations continue to decrease under the RCP 8.5 scenario. The Gulf of Maine and Georges Bank rely on *C*. *finmarchicus* as a crucial link in the food web, transferring energy from phytoplankton through the marine food web and into vital fisheries species, such as larval cod and haddock^6^. *C*. *finmarchicus* is the primary prey of the North Atlantic right whale (*Eubalaena glacialis*), which is endangered with an estimated 526 individuals cataloged by the North Atlantic Right Whale Consortium in 2014^73^. North Atlantic right whales have shown extremely slow recovery since the 1931 commercial fishing ban, possibly indicating that fluctuating food availability is contributing to their limited population growth^7, 74^, although incidental anthropogenic interference (e.g. entanglements, ship strikes) is still a significant source of mortality^75^. These whales require ~500 kg of *C*. *finmarchicus* per day to sustain themselves^76^. As a result, calving success is correlated with *C*. *finmarchicus* concentration even at regional scales^9^ despite right whales requiring small (10 s of m), extremely dense patches of *C*. *finmarchicus* ^77^. Although our model did not resolve these smaller spatial scales, we do show that the large-scale conditions for *C*. *finmarchicus* are going to become less favorable under climate change. These conditions are likely to negatively affect *C*. *finmarchicus* size as well as density^78–80^, potentially having a severe negative effect on right whales and the rest of the food web.

Despite these declines, we still expect *C*. *finmarchicus* to be present in the Northeast U.S. Shelf year round, supporting the results of the GAM used by Villarino *et al*.^35^. This contrasts with the work of Reygondeau and Beaugrand^38^, who projected that *C*. *finmarchicus* would be absent (probability of occurrence <0.1) by 2050–2059 under the IPCC B2 scenario. This discrepancy could be due to a variety of reasons, including the lower spatial resolution of the climate model analyzed in that study (MPI-ECHAM4, 1° × 1°), the lack of seasonal temporal resolution, different *C*. *finmarchicus* life cycle stages used to train the model, and structural differences of the statistical models. Each of these projections used different climate scenarios, and Reygondeau and Beaugrand used the least severe scenario of the three, indicating that climate scenarios are unlikely to be the cause for the discrepancy between the models.

In other niche models^20, 35, 38, 81^, SST was determined to be an extremely important component in determining probability of occurrence. However, SST was the only variable we used that was deemed insignificant by the GAM. This is due to our inclusion of BT, without which SST is highly significant. This could indicate that BT is a better variable to use in modelling *C*. *finmarchicus*, or that dynamics are different when attempting to model the entire North Atlantic instead of just the Northwest Atlantic studied here. Additionally, BT could be a more important factor in determining density, but likelihood of occurrence can be modelled using only surface variables. This model is only a proxy for the different effects of changing conditions, so the exact physiological and life history mechanisms being disrupted by climate change are uncertain. Because depth is static, BT is the most important variable in our study driving the declines of *C*. *finmarchicus* with climate change. Increasing temperature can affect *C*. *finmarchicus* populations in a variety of ways including decreasing body size^78, 79^, egg production^23^, or available nutrients and dissolved oxygen^48^.

Overall, we are projecting substantial decreases of *C*. *finmarchicus* in the Northeast U.S. Shelf due to anthropogenic climate change by the end of the century. Mitigation is likely to only affect the magnitude of these changes, not the likelihood. This could have profound impacts on the North Atlantic right whale and many other economically and socially important species that are dependent on *C*. *finmarchicus*. However, more detailed projections involving species interactions and physical effects not considered here are still required in order to best assess the threat to *C*. *finmarchicus* on the Northeast U.S. Shelf.

## Electronic supplementary material

Supplementary Information
