# Supplementary material for: Projecting the effects of climate change on Calanus finmarchicus distribution within the U.S. Northeast Continental Shelf
Source: Sci Rep. 2017 Jul 24;7:6264. doi: 10.1038/s41598-017-06524-1 (PMC5524788; doi:10.1038/s41598-017-06524-1)

Projecting the effects of climate change on *Calanus finmarchicus* distribution within the U.S. Northeast Continental Shelf

Brian D. Grieve[1,2*], Jon A. Hare[3], Vincent S. Saba[4]

1. NOAA NMFS Northeast Fisheries Science Center, Narragansett, RI 02882, USA
2. Integrated Statistics, Woods Hole MA 02543, USA
3. NOAA NMFS Northeast Fisheries Science Center, Woods Hole, MA 02543 USA
4. NOAA NMFS Northeast Fisheries Science Center, Geophysical Fluid Dynamics Laboratory, Princeton University Forrestal Campus, Princeton, NJ 08540, USA

* Corresponding author: brian.grieve@noaa.gov

Figure S1: Sampled regional mean *Calanus finmarchicus* density over bi-monthly seasonal (a) and annual (b) time scales. The black line is the overall mean. Error bars indicate standard error of the mean of individual samples in each region.

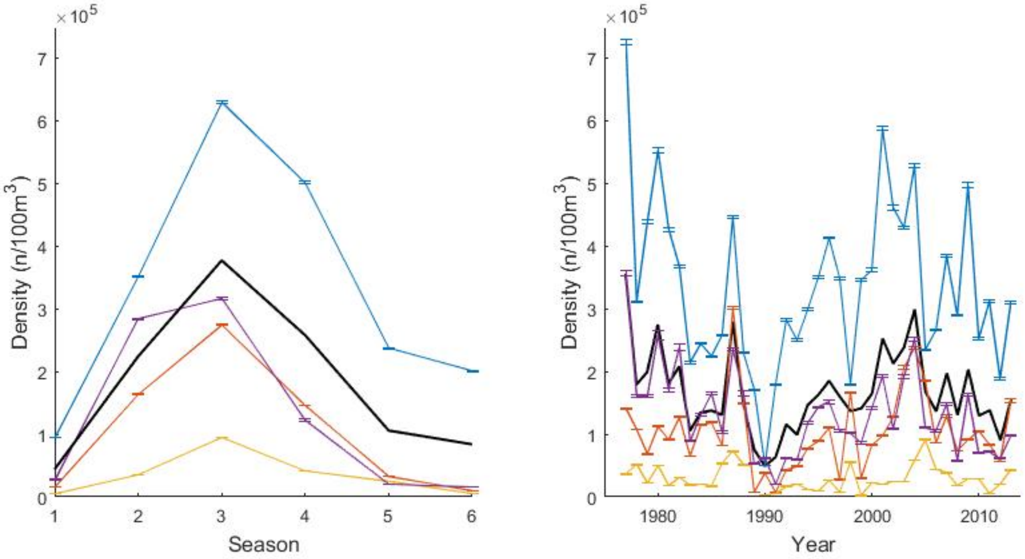

Supplementary Figure S2: *C. finmarchicus* density sampled by NOAA from 1977-2013. Figure was created by the first author in MATLAB v2015b (https://www.mathworks.com/products/matlab.html) using the package ‘M_Map’ v1.4h (https://www.eoas.ubc.ca/~rich/map.html).

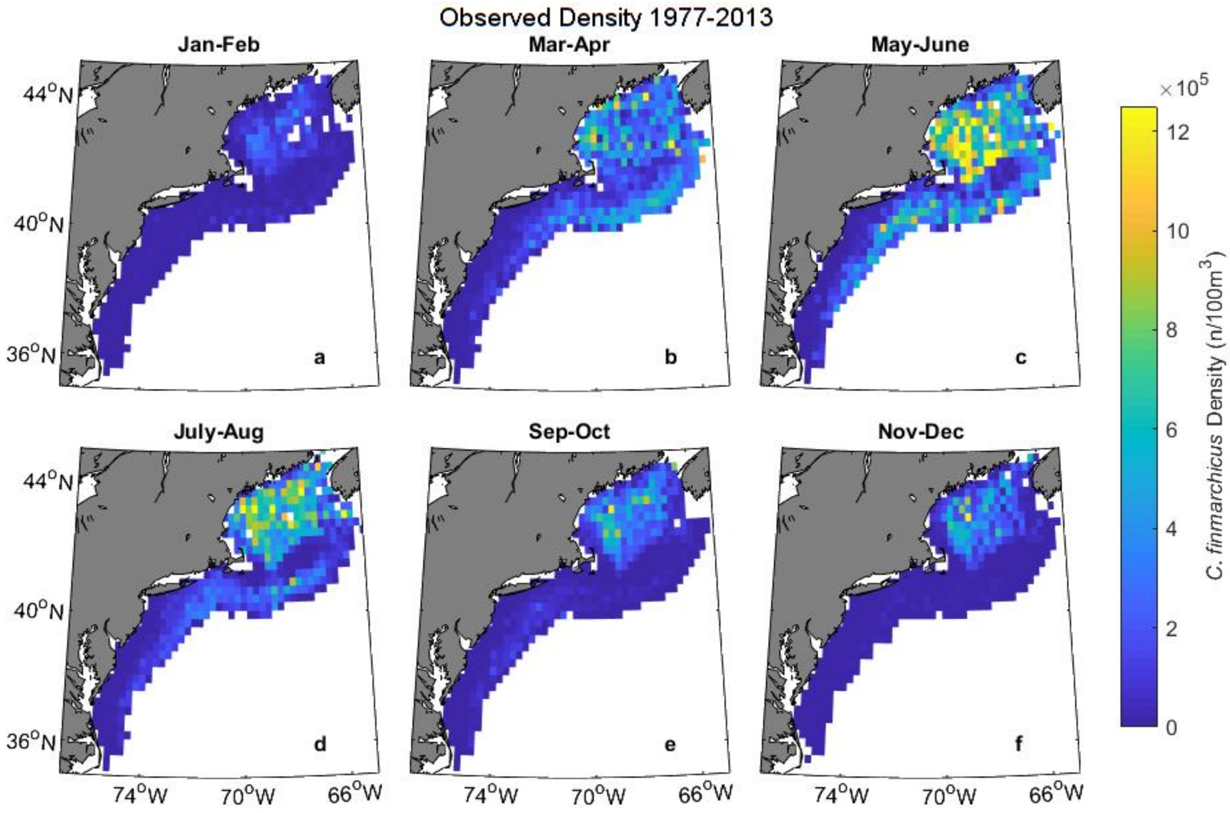

Supplementary Figure S3: Residuals of fitted GAM. The left figure shows deviance residuals plotted against the linear predictor. The right figure shows a variogram of pearson residuals against distance (degrees).

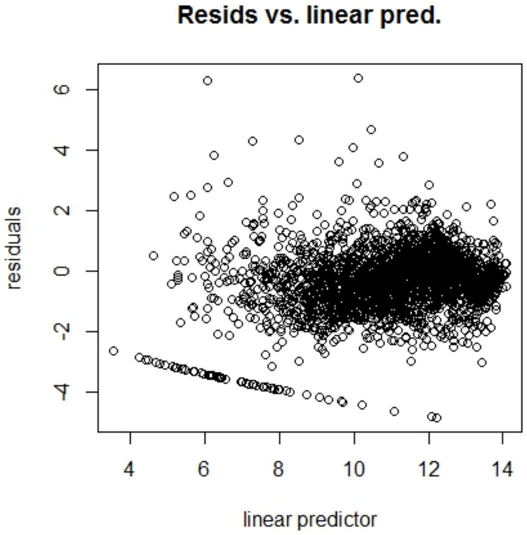

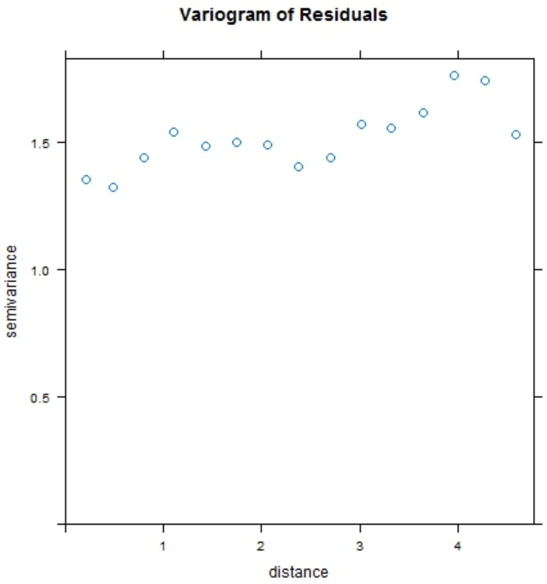

Supplementary Figure S4: Differences in *C. finmarchicus* density between the observations and the RCP 8.5 model projections over the sample time period, presented as a percentage of the observations. A positive value indicates that the model projected higher than the observations. Figure was created by the first author in MATLAB v2015b (https://www.mathworks.com/products/matlab.html) using the package 'M_Map' v1.4h (https://www.eoas.ubc.ca/~rich/map.html).

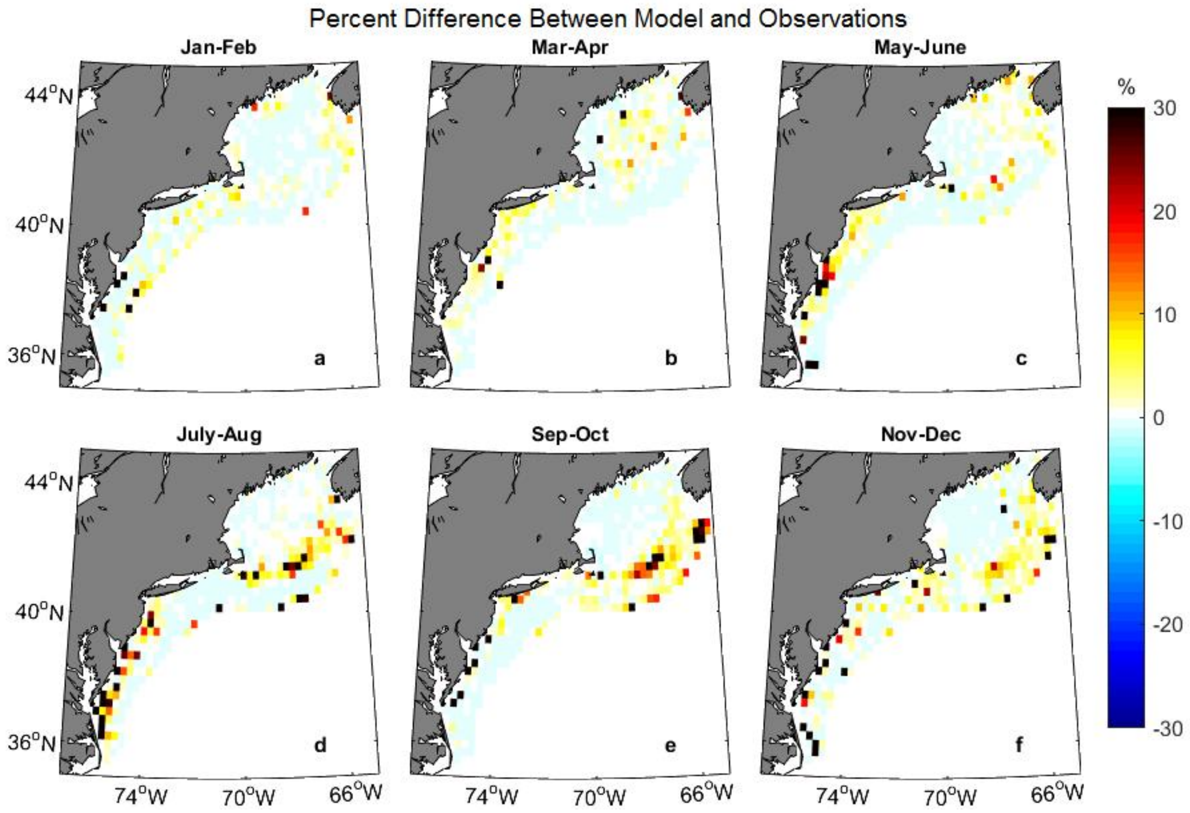

Supplementary Figure S5: *C. finmarchicus* density during the 2041-2060 period under RCP 4.5. Figure was created by the first author in MATLAB v2015b (https://www.mathworks.com/products/matlab.html) using the package ‘M_Map’ v1.4h (https://www.eoas.ubc.ca/~rich/map.html).

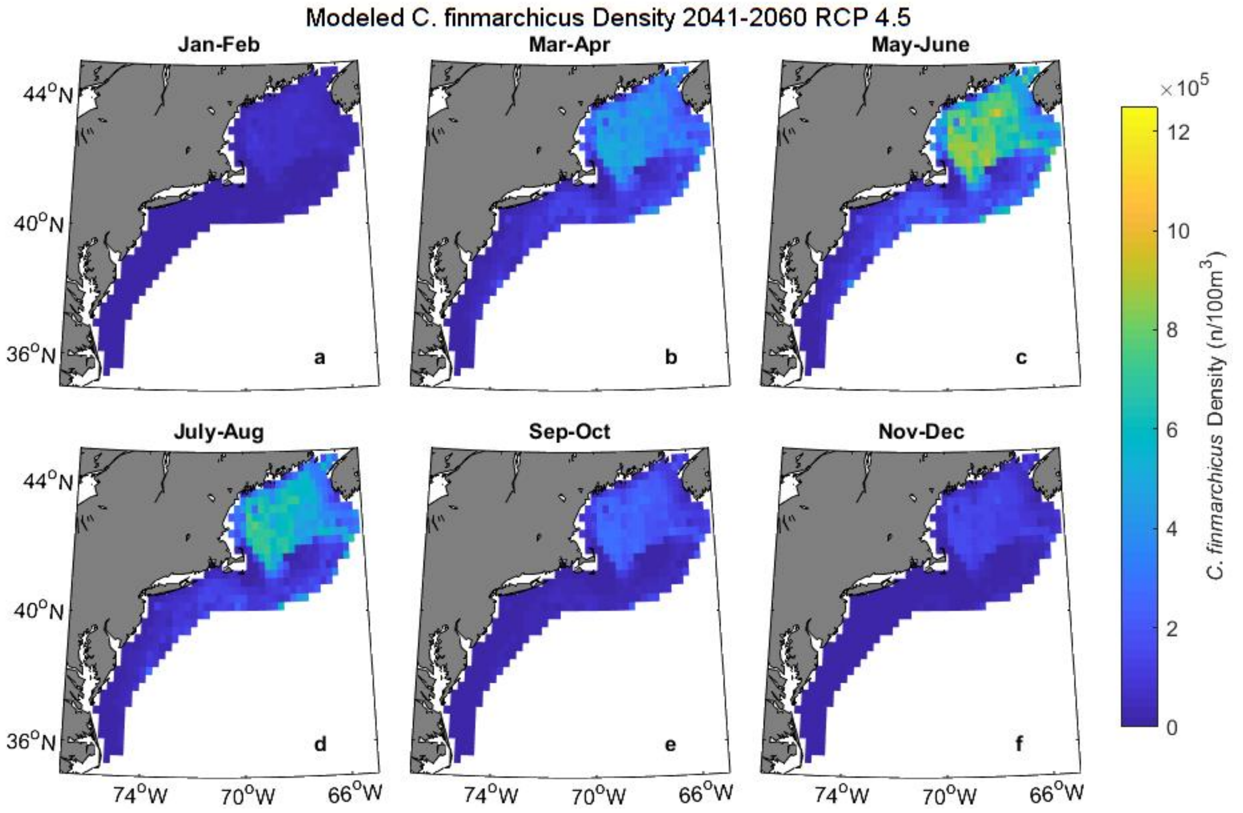

Supplementary Figure S6: *C. finmarchicus* density during the 2041-2060 period under RCP 8.5. Figure was created by the first author in MATLAB v2015b (https://www.mathworks.com/products/matlab.html) using the package ‘M_Map’ v1.4h (https://www.eoas.ubc.ca/~rich/map.html).

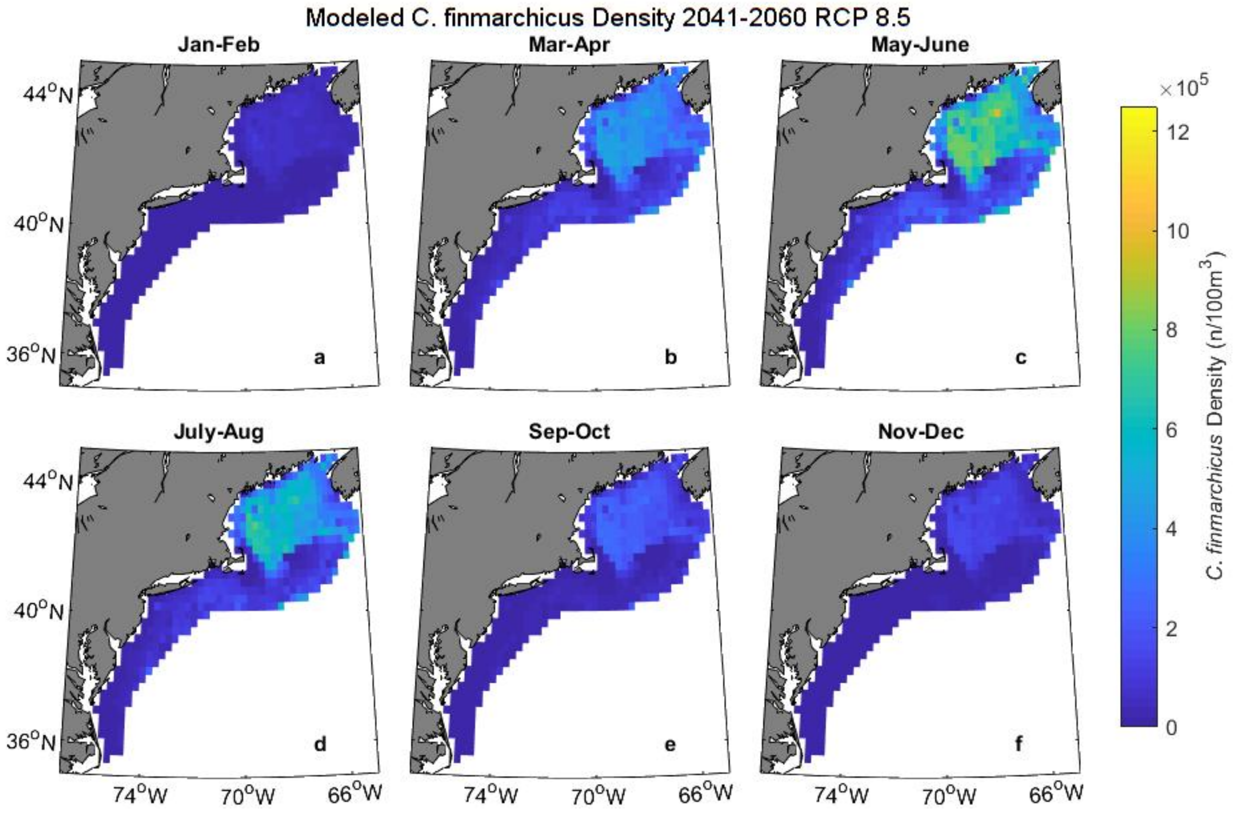

Supplementary Figure S7: *C. finmarchicus* density during the 2081-2100 period under RCP 4.5. Figure was created by the first author in MATLAB v2015b (https://www.mathworks.com/products/matlab.html) using the package ‘M_Map’ v1.4h (https://www.eoas.ubc.ca/~rich/map.html).

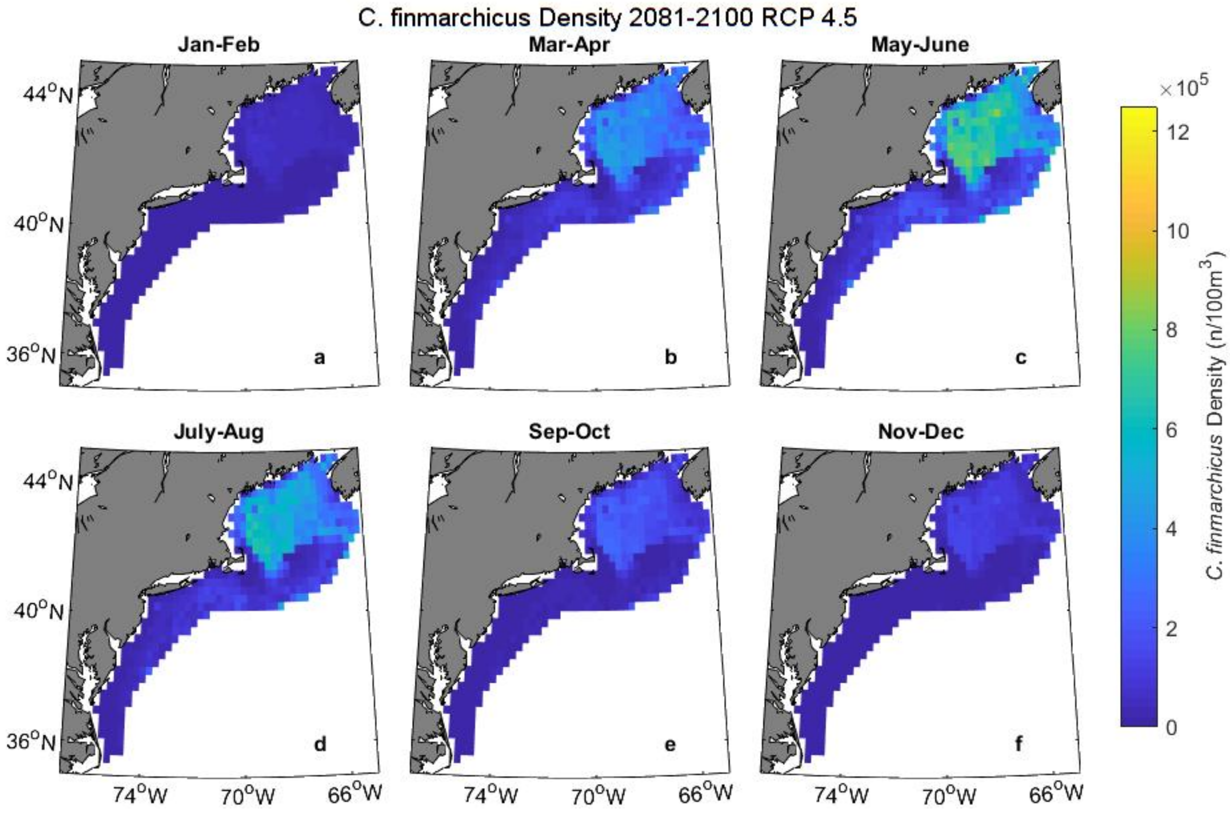

Supplementary Figure S8: *C. finmarchicus* density during the 2081-2100 period under RCP 8.5. Figure was created by the first author in MATLAB v2015b (https://www.mathworks.com/products/matlab.html) using the package ‘M_Map’ v1.4h (https://www.eoas.ubc.ca/~rich/map.html).

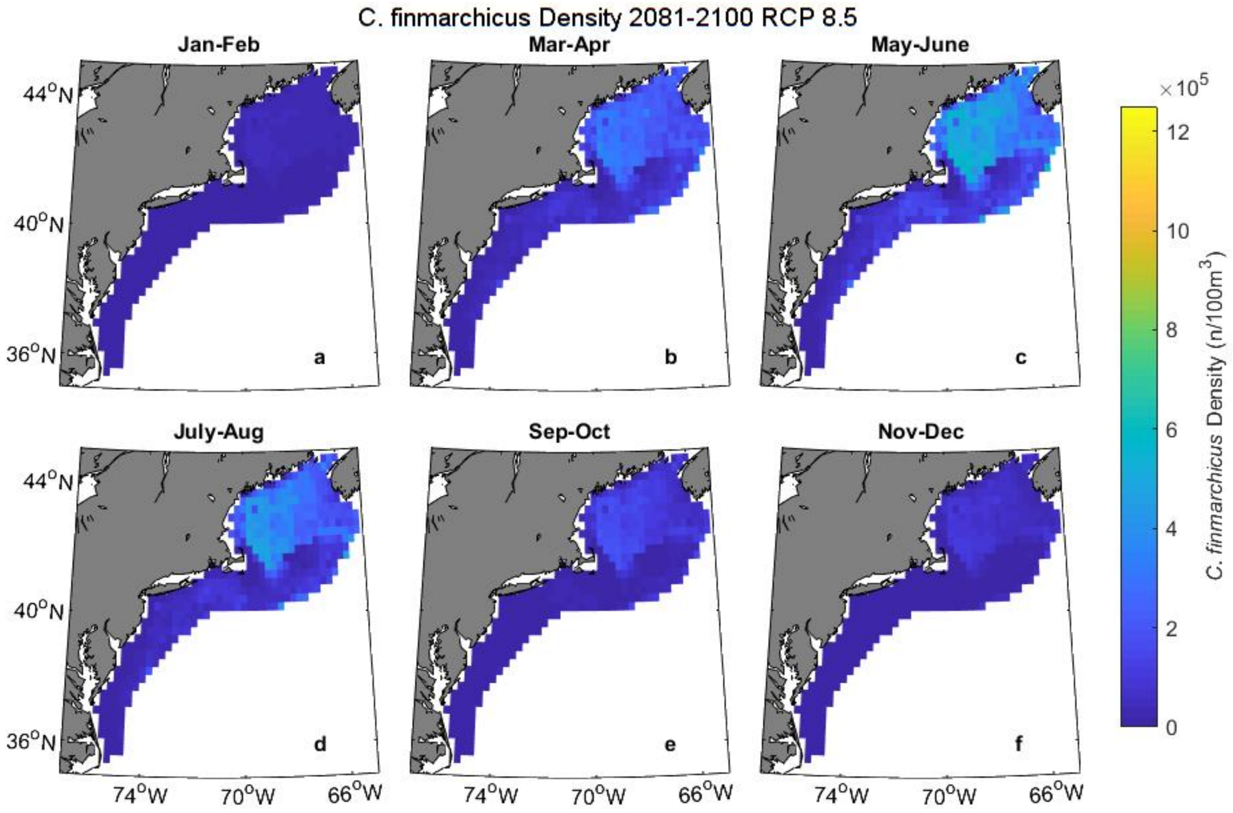

Supplement: Supplementary file 1 — Supplementary Information [file 41598_2017_6524_MOESM1_ESM.pdf]
